# Extramedullary versus intramedullary tibial cutting guides in megaprosthetic total knee replacement

**DOI:** 10.1186/1749-799X-7-33

**Published:** 2012-10-02

**Authors:** Vikas Karade, B Ravi, Manish Agarwal

**Affiliations:** 1 Department of Mechanical Engineering, Indian Institute of Technology Bombay, 400076 Mumbai, Maharashtra, India; 2 P.D Hinduja National Hospital & MRC, Veer Savarkar Marg, Mahim, 400076 Mumbai, India

**Keywords:** Megaprosthetic knee replacement, Cutting guide, Tibial component, Tibial cut, Statistical analysis

## Abstract

**Background:**

In a standard total knee replacement, tibial component alignment is a key factor for the long term success of the surgery. The purpose of this study is to compare the accuracy of extramedullary and intramedullary tibial cutting guides used in indigenous and imported implants respectively, in positioning of the tibial components in megaprosthetic knee replacements.

**Methods:**

A comparative study of the accuracy of extramedullary and intramedullary tibial cutting guides was carried out in 92 megaprosthetic knee replacements for distal femoral tumors. For the proximal tibia cut for tibial component placement, an extramedullary guide was used in 65 patients and an intramedullary guide was used in 27 patients. Tibial component alignment angles were measured in postoperative X-rays with the help of CAD software.

**Results:**

There was more varus placement in coronal plane with extramedullary cutting guide (−1.18 +/− 2.4 degrees) than the intramedullary guide (−0.34 +/− 2.31 degrees) but this did not reach statistical significance. The goal of 90 +/− 2 degrees alignment of tibial component was achieved in 54% of patients in the extramedullary group versus 67% in the intramedullary group. In terms of sagittal plane alignment, extramedullary guide showed less accurate results (2.09 +/− 2.4 degrees) than intramedullary guide (0.50 +/− 3.80 degrees) for tibial component alignment, though 78% of patients were aligned within the goal of 0–5 degrees of tibial slope angle in extramedullary group versus 63% in intramedullary group. The mean error in the measurements due to rotation of the knee during taking the X-rays was less than 0.1 degrees and distribution of the X-rays with the rotation of knee was similar in both the groups.

**Conclusions:**

Overall, in megaprosthetic knee replacement intramedullary guides gave more accurate results in sagittal plane and exhibited similar variability as of extramedullary guides in coronal plane.

## Background

In a standard total knee replacement (TKR), the proximal tibia has to be cut for placement of the tibial component base plate. The tibial component alignment is a key factor for the long term success of the surgery [1-3]. This has not been well documented for megaprosthtic knee replacement surgery. Though the implant is constrained and a stemmed tibial component is used, accurate placement may have a role in long term survival of the implant.

In megaprosthetic knee replacement, the tibial component stem is required to be parallel to mechanical axis of tibia in both coronal and sagittal plane. The mechanical axis is defined as the line connecting the center of femoral head and center of talus bone [4]. The mechanical axis of the tibia is nearly parallel to the anatomical axis which is the line connecting midpoints of outer cortical diameter at around 5 and 15 cm distal to the knee joint [4,5]. The anatomical axis runs along the tibial canal as well. In indigenous implant system (ResTOR knee system), the tibial component stem is 10cm in length and 10mm in diameter. The stem is not long enough to engage the narrowest portion of the tibial intramedullary canal for self-alignment along the anatomical axis. As the tibial component base plate aligns itself along the cut plane, an accurate alignment of the cut plane with respect to the anatomical axis of the bone becomes very important. The cut should be perpendicular to the anatomical axis of the tibia. Extramedullary and intramedullary cutting guides are used predominantly to obtain accurate tibial cuts. Indigenous implant system (ResTOR knee system Sushrut, India) comes with an extramedullary cutting guide whereas the imported implant system (GMRS knee system, Stryker, USA) comes with an intramedullary cutting guide. Both guides have an alignment rod which has to be first placed parallel to the anatomical axis. Then a saw blade is guided to make a cut perpendicular to the rod. Both types of conventional guides use anatomical landmarks to position their alignment rod parallel to the anatomical axis of tibia [6]. Besides bone landmarks, use of palpable tendons as reference has also been reported [7,8]. Both cutting guides have specific advantages and limitations. Extramedullary guides maybe easier to use due to the long familiarity with standard knee replacements. Intramedullary guides have direct access to tibial canal and hence the anatomical axis, and are believed to be more accurate. There is however, a lack of consensus on which of the cutting guides gives better accuracy of cuts. A few studies have shown that intramedullary guides give more accurate cutting plane alignment than extramedullary guides in total knee arthroplasty [2,3,9]. No study appears to have been reported to compare the tibial cutting guides in megaprosthetic knee replacement for distal femoral tumor. The research objective of this study is to compare the accuracy of intramedullary and extramedullary guides used in the two implant systems as mentioned earlier for tibial resection.

## Methods

### Patients and surgical technique

Ninety two X-rays of patients who had undergone distal femur megaprosthetic knee replacement surgery between August 2004 to August 2009 were studied. The surgeries were carried out by one of the authors (Dr. Manish Agarwal) at various institutes. For this kind of a retrospective study, no ethical clearance is required at our institutes. The patients included 63 male and 29 female, with a mean age of 23.65 years. The number of right and left knee operated were 46 each. To cut and position the tibial component an extramedullary or intramedullary cutting guides were used. The cutting guides were linked to the type of the implant system used for the surgery. Imported implant system (GMRS total knee system, Stryker, USA) was used for patients who could afford the same. An indigenous implant system (RESTOR total knee system, Sushrut Adler, India) was used for all others. Extramedullary cutting guide was used in a group of patients (extramedullary group, n=65) who preferred Indigenous implant system. Intramedullary cutting guide was used in a group of patients (intramedullary group, n=27) who preferred imported implant system. The baseline patient characteristics are shown in Table 1.

**Table 1:** Baseline patient characteristics

		**Extramedullary group**	**Intramedullary group**	**Total**
n (number of patients)		65	27	92
Mean age in years (S.D.)		22.42 (9.78)	24.89 (11.09)	23.14 (10.18)
Gender	Male	43	20	63
	Female	22	7	29
Number of Left/Right legs	Left	31	15	46
	Right	34	12	46

*S*.*D*. standard deviation.

**Figure 1:**
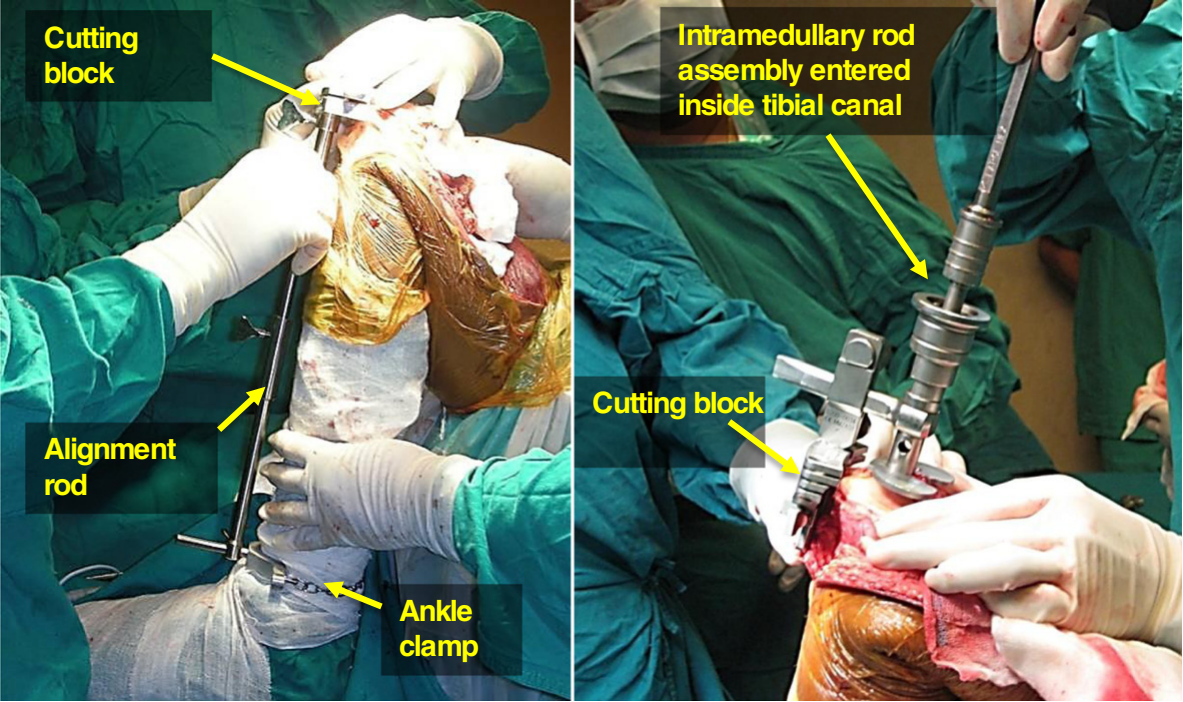
**Extramedullary guide (left) andintramedullary guide (right).**

The extramedullary cutting guide has three components: a clamp around the ankle, a cutting block for proximal tibial cut and a connecting rod between these two which is positioned along the shin and just anterior to it, with rotation also being set referencing the medial third of tibial tubercle and transmalleolar axis. The proximal portion of the alignment rod (Figure 1) was positioned over the medial third of the tibial tubercle. For anterior posterior alignment, the anterior surface of tibia was used as a reference. These adjustments were based solely on visual judgment. The cutting block with a posterior slope of zero degree was assembled to the alignment rod and fixed to the bone by pins hammered to anterior surface of proximal tibia. The assembly ensures that slit opening of the cutting block, which guides the cutting saw blade, was perpendicular to the alignment rod. Tibial stylus was used to determine the exact bone resection thickness. After cutting block fixation, the bone was cut with the help of an oscillating saw.

In the intramedullary group, an entry hole was created in the articular surface near the base of the anterior tibial spine using a six millimeter drill. This position is usually located in anterior one-third of the tibial articular surface. The entry hole was reamed progressively till a tight canal fit was obtained (10–21 mm). The medullary contents were decompressed by suction. Reamers were used to size the canal and intramedullary guide assembly was inserted using the appropriate diameter stem. Rotation alignment was referenced to tibial tubercle. The cutting block with a posterior slope of zero degree was assembled to the intramedullary guide assembly. The design of the guide ensures that the cutting block lies over the anterior portion of tibia, and the slit opening of the cutting block is perpendicular to the intramedullary rod. A stylus was used to determine the thickness of bone resection. The cutting block was secured to the tibia with pins. Then a cutting saw blade was inserted through the slit opening of the cutting block and the tibial bone slice was cut.

### Measurement technique and X-ray validation

**Figure 2:**
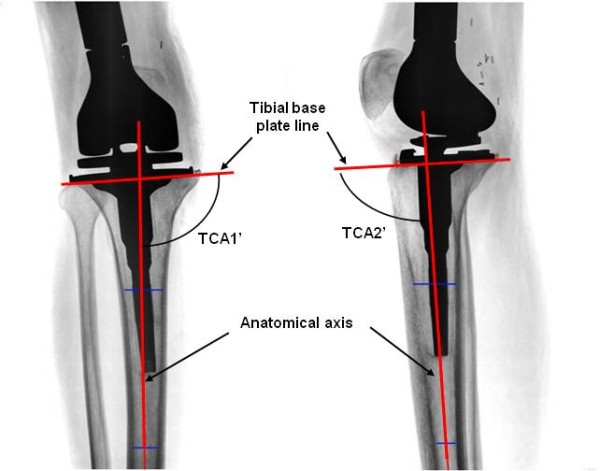
**Measurement of tibial componentangle in coronal viewand sagittal view onX-ray.**

Each patient was evaluated using postoperative X-rays of their tibial portion: both coronal and sagittal view. The alignment angles of tibial component in both the views were measured and the error was calculated. The assessor who performed the measurement was blinded to which cutting guide was used intraoperatively. All X-ray images (in JPEG format) were imported in CAD software (SolidWorks 2008) and lines were drawn to mark the anatomical axis line as well as tibial base plate line (Figure 2). The postoperative measurements involved angles which represent the error in tibial component alignment. The tibial component angle in coronal view, TCA1’ was defined as the angle between tibial base plate line and anatomical axis, measured from medial side of tibia. Similarly in sagittal view, another tibial component angle TCA2’ (tibial slope) between tibial base plate line and anatomical axis, was measured from anterior side. The error in tibial component alignment in coronal view was defined as TCA1 obtained by subtracting 90° from TCA1’. Similarly in sagittal view the error was defined as TCA2 obtained by subtracting 90° from TCA2’. A negative value of TCA1 indicates varus placement i.e., lateral region of cut is lower than the medial region. Similarly, positive TCA1 value indicates valgus placement. A negative value of TCA2 indicates anterior slope i.e., anterior region of the cut is lower than the posterior region. Positive value indicates a posterior slope of tibial component. Figure 2 shows a valgus placement of tibial cut with a small anterior slope. The optimal value for angle TCA1 is 0 +/− 2° and that for the angle TCA2 is 0-5°. Alignments of the tibial component that fell within the above range were considered to be acceptable.

**Figure 3:**
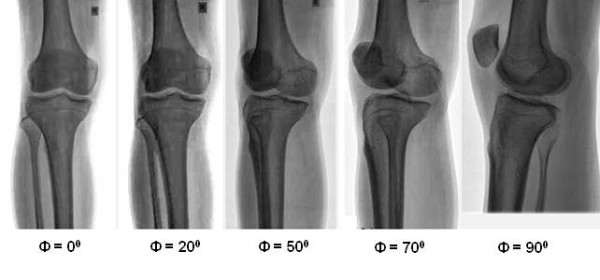
**Benchmark X-rays taken atpredetermined rotation angles.**

**Figure 4:**
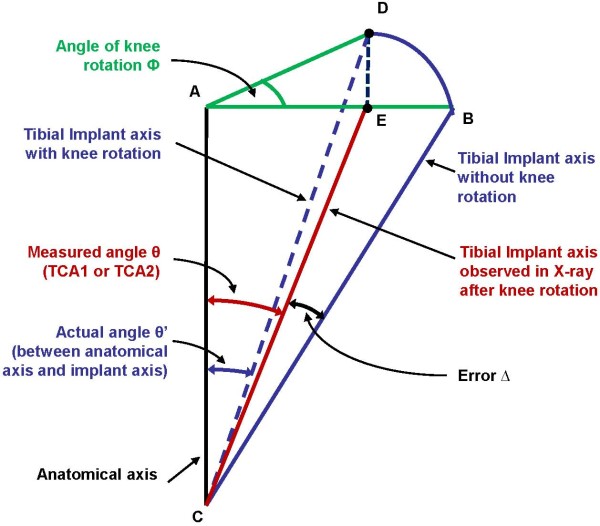
**Schematic showing an angularerror (Δ) in themeasurement of tibial componentangle (θ)**, **occurring due to kneerotation (Ф)**

**Figure 5:**
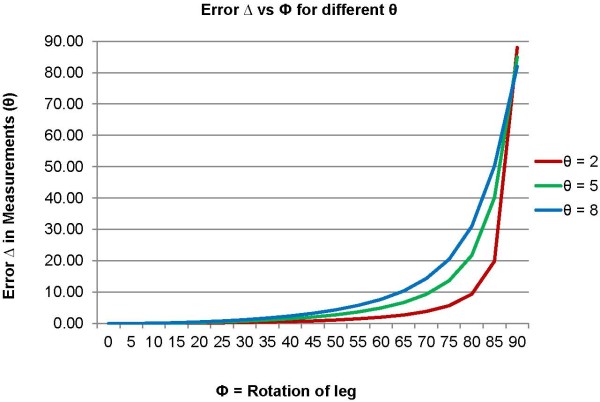
**Variation of angular error(Δ) with knee rotationangle Ф for threedifferent angular measurements (θ)of tibial component angle.**

It was observed that there is a rotation of the knee along the axis toward lateral side in a few coronal view X-rays. Similarly, in a few sagittal view X-rays also there is knee rotation towards the medial side. This rotation can include an error in the angular measurements of TCA1 or TCA2. This error (Δ) depends upon the rotation angle (Ф) of the knee as well as the angular measurements (θ) of TCA1 or TCA2. To find the error in the angles TCA1 and TCA2, all the postoperative X-rays were first compared to benchmark X-rays (Figure 3) which were taken at predetermined angles (0^0^, 20^0^, 50^0^, 70^0^ and 90^0^) of knee rotation. The benchmark X-ray at 0^0^ belongs to a true coronal plane of knee and was taken by keeping the leg position in such a way that patella was facing upwards. The X-ray at 90^0^ belongs to a true sagittal plane of knee. X-rays belonging to 20^0^, 50^0^, 70^0^ and 90^0^ were measured with respect to the 0^0^ position and taken by rotating the knee towards lateral side. The angles were measured at the feet region with the help of goniometer. The comparison of postoperative X-rays with these benchmark X-rays was based upon some unique observations in the benchmark X-rays as summarized in Table 2. Two different sets of such benchmark X-rays were used to confirm the comparison results. This comparison helped in categorizing the postoperative X-rays according to the rotation of the knee included in them. It was observed that only a few postoperative X-rays were having a knee rotation close to 20^0^. The majority of X-rays were taken in the correct coronal or sagittal view. Hence, a few coronal view X-rays were categorized as having an angle of 20^0^ and a few sagittal view X-rays were categorized as having an angle of 70^0^. The error Δ in the measurements θ of TCA1 or TCA2 in each X-ray was then measured by equation (1). A relation between an angular error (Δ) in measurement of tibial component angle (θ) and knee rotation (Ф) is shown in Figure 4. The variation of the angular error Δ with respect to the knee rotation Ф for three different angular measurements θ is shown in Figure 5.

(1) $$▵=\theta ’-\theta =\left\{\tan^{-1}\left(\tan\;\theta /\cos\;Ф\right)\right\}-\theta$$

where,

TKR: Total knee replacement; TCA: Tibial component angle; TKA: Total knee arthroplasty; IM: Intra medullary; θ’: actual angle between anatomical axis and tibial implant axis; θ: angular measurements of tibial component angle (TCA1 or TCA2); Δ: angular error in measurements of θ; Ф: rotation of the knee.

**Table 2:** **Unique features observed inbenchmark X-rays of kneetaken at different angles**

**Angle**	**Unique feature observed**
0^0^	Fibula can be seen distinctly and patella is at the center covering the knee joint
20^0^	Fibula is partially covered by tibia and patella is not at the center
50^0^	Fibula is totally covered by anterior part of tibia and patella is not at center
70^0^	Fibula is totally covered by posterior part of tibia and patella is not at center
90^0^	Fibula and patella can be seen distinctly

A power analysis showed that with the present sample size, a mean difference of 1.5° can be detected with 86% probability (alpha = 0.05, one sided comparison of two independent means). Outlier data detection was performed using Grubbs' test assuming that the errors are normally distributed (central limit theorem). Two null hypotheses were stated: (i) the intramedullary group shows less mean error than extramedullary group in coronal view and (ii) the intramedullary group shows less mean error than extramedullary group in sagittal view. Statistical significance of the difference in mean errors was calculated using independent t-test for two independent samples. Statistical significance of the difference in the percentage of correct tibial alignment was calculated using Chi-square test for two independent samples. Statistical significance of the difference in terms of surgeon, gender of the patient, number of left/right leg and number of X-rays with knee rotation was also determined using Chi-square test for two independent samples. The mathematical calculations and the statistical analysis were performed using Microsoft Excel 2007 and Minitab statistical software respectively. A p value greater than 0.05 was taken as statistical significance.

## Results

No outlier in the data was detected and all the 92 X-rays were used for error measurement and analysis (Grubb’s test). There was no statistical difference between the two groups in terms of age, gender of the patient and leg operated (p ≫ 0.05, Chi square test). Each surgeon was involved in a comparable number of both extramedullary assisted and intramedullary assisted surgeries. No significant difference was found between the two groups with respect to surgeon involved in surgeries. The null hypotheses were examined by one sided independent t-test. The mean value of tibial component alignment error in coronal view i.e. TCA1 in extramedullary group was −1.18 +/− 2.40° and in intramedullary group it was −0.34 +/− 2.31°. This difference was not statistically significant (for alpha = 0.05). Therefore, we reject the first null hypothesis. In the sagittal view, extramedullary group showed a value of the mean TCA2 as 2.09 +/− 2.40° compared to 0.50 +/− 3.28° for the intramedullary group. This was a statistically significant difference (p < 0.05). Hence, we accept the second null hypothesis. The tibial component angle in the coronal view (TCA1) fell within the optimal range in 54% cases of extramedullary group, while the results in intramedullary group showed 67% of cases in the optimal range. This difference was not statistically significant. The extramedullary group showed 25% cases having valgus placements (in other words, 75% varus placements) compared to 44% in intramedullary group. This difference was also not statistically significant. Regarding tibial component angle in sagittal view (TCA2), in extramedullary group 78% cases were in the optimal range while in intramedullary group only 63% cases fell into the optimal range. The difference was not statistically significant. The extramedullary group showed 88% of cases having posterior slope (12% anterior) compared to 67% in the intramedullary group. This difference was statistically significant (p < 0.05). In the extramedullary group, 29% of coronal view X-rays had nearly 20^0^ rotation of knee compared to 33% in the intramedullary group. This difference is not statistically significant. The extramedullary group had 38% of sagittal view X-rays with 70^0^ (instead of 90^0^, i.e. true sagittal plane) compared to 48% intramedullary group. The difference was again not statistically significant. The mean error in the measurements of TCA1 and TCA2 due to knee rotation while taking coronal and sagittal view X-rays respectively was less than 0.1 degrees. The results are summarized in Table 3.

**Table 3:** **Results for postoperative measurementsin both groups**

			
*Postoperative measurements in coronalview*			
	Extramedullary	Intramedullary	Significance
Mean TCA1 +/− S.D. in degrees	−1.18 +/− 2.40	−0.34 +/− 2.31	p = 0.06
Number of X-rays within optimal range (percentage)	35 (54)	18 (67)	p = 0.25
Number of X-rays within valgus alignment (percentage)	16 (25)	12 (44)	p = 0.07
Mean error Δ in degrees	0.05	0.04	p = 0.65
Number of X-rays tends to 20^0^ leg rotation (percentage)	19 (29)	9 (33)	p = 0.70
*Postoperative measurements in sagittalview*			
	Extramedullary	Intramedullary	Significance
Mean TCA2 +/− S.D. in degrees	2.09 +/− 2.40	0.50 +/− 3.28	p = 0.01
Number of X-rays within optimal range (percentage)	51 (78)	17 (63)	p = 0.16
Number of X-rays within posterior slope (percentage)	57 (88)	18 (67)	p = 0.03
Mean error Δ in degrees	0.07	0.05	p = 0.46
Number of X-rays tends to 20^0^ leg rotation (percentage)	25 (38)	13 (48)	p = 0.38

TCA1: error in tibial component angle measured in coronal view.

TCA2: error in tibial component angle measured in sagittal view.

S.D.: Standard Deviation.

Error Δ: Angular error in measurements (θ) of TCA1/TCA2 occurring due to knee rotation (Ф).

Statistical significances were set at p < 0.05.

## Discussion

In this study, the extramedullary group exhibited more mean error in coronal plane alignment of tibial component (−1.18 +/− 2.4°) compared to intramedullary group (−0.34 +/− 2.31°). However, this difference was not statistically significant (p = 0.06). The percentage of the optimal cases in both groups was also not significantly different. In the extramedullary group, the percentage of varus placements (75%) was three times the percentage of valgus placements (25%). The reason for this may lie in the distal alignment of the extramedullary rod. The proximal part of the extramedullary rod was aligned over the medial third of tibial tubercle and the distal part was aligned over the center of ankle. However, the mechanical axis (hence the anatomical axis parallel to it) runs through the mid-point of talus bone which usually lies on the medial side of ankle center. If this fact is not considered then the alignment rod will not be parallel to mechanical axis, but will be rotated toward the lateral side resulting in most of the final tibial component placement to be in varus. However, when the percentage of the varus cuts in the extramedullary group were compared to that in the intramedullary group, then no statistically significant difference was found. A few researchers have reported better accuracy using extramedullary guides when the distal alignment of extramedullary rod is taken as three millimeters medial to the mid-point of ankle [3]. For intramedullary guides, one of the reasons mentioned by surgeons for inaccuracy is the instability of intramedullary rod (IM rod). The intramedullary guide relies on the parallelism of IM rod and tibial canal (and hence the anatomical axis). Usually the IM rod is thinner than tibial canal, which may cause a tilt of the rod inside the canal and hence a tilt in the cutting block alignment. The entry point position is also a key factor. The ideal entry point position is on the tibial articular surface corresponding to the proximal continuation of tibial canal [10]. For example, in tibia with obvious varus deformity, the entry point needs to be more lateral than usual. Hence, an ideal entry point position should be preoperatively determined with the help of X-rays.

The sagittal alignment (TCA2) of a replaced knee is kinematically important because most of the knee motion occurs in this plane [5]. In this context, intramedullary guide showed better accuracy than with an extramedullary guide (p < 0.05). In this study there was no significant difference in the number of cases falling in the optimal range of 0–5°. The reason for both these observations lies in the tendency of extramedullary guide to give a posterior slope in the tibial cut. This tendency makes the mean tibial component angle in sagittal view (TCA2) to deviate away from the desired 0° angle. It also increases the number of cases having a posterior slope. Indeed, there were 88% cases with posterior slope in extramedullary group compared to 67% in intramedullary group (p < 0.05). To avoid an undesirable anterior slope, surgeons try to give a posterior slope by moving the distal part of alignment rod a little away from the anterior tibial surface. This explains the tendency of the extramedullary guide to give a posterior slope in the tibial cut. In the intramedullary guide, once the IM rod is inserted inside the tibial canal, there is no possibility of adjustment of the tibial slope by moving the rod. The surgeon can only use different cutting blocks (giving 3-5° slope). These results for sagittal plane are in contrast to the results presented in a study performed by Cashman JP et al., where extramedullary guides showed better accuracy, for total knee arthroplasty (TKA) surgery [11]. A prospective study performed by Maestro et al. on TKA surgery cases showed no statistical difference in sagittal plane positioning of the tibial component [2].

Extramedullary guides can be used in both deformed and non-deformed bones but are sensitive to surgical performance, while intramedullary guides rely on non-deformed tibial canals. Extramedullary guides are easy to use and can be employed for patients with low fat accumulation over their leg. Fat embolism and intramedullary fracture are considered as drawbacks for using intramedullary guides. The principle of using both the cutting guides is to make their alignment rod parallel to anatomical axis by using landmarks and then cut perpendicular to the alignment rod. The principle is perfect but the design and the procedure need some modification and improvements. To get a proper guidance for the center of the ankle, the design of extramedullary guide may need to be modified and used with proper preoperative planning. A marker can be placed externally over the ankle center which can be determined intraoperative by X-rays. This external marker can be used to align the distal part of alignment rod of the extramedullary guide. The design of the intramedullary guide needs to be improved in such a way that while making IM rod parallel to the tibial canal there should be no error. The IM rod should not wobble inside the canal. To ensure a correct position of the entry point, a preoperative plan must be made with the help of X-rays. Finally, while using either guide, the lower limb must be rigidly fixed to avoid disturbances during cutting block alignment, cutting block fixation and cutting. A combination cutting guide can be developed where the alignment of IM rod of the intramedullary guide can be cross-checked with an external alignment rod of extramedullary guide.

Rotation of knee while taking an X-ray also incorporates an error which was however, found to be very small (< 0.1^0^). In this study, a few postoperative X-rays had a rotation of knee between 0^0^ and 20^0^, when compared to the benchmark X-rays. Equation 1 shows that larger the measurement angle θ more is the error included by a knee rotation of the same angle Ф. It is shown that the error Δ is very small for smaller knee rotation angles Ф and rapidly increases as Ф rises to 90^0^ (Figure 5). The distribution of X-rays with knee rotation was similar in both extramedullary and intramedullary group. A standard method should be devised to take a correct coronal view and sagittal view X-rays [12]. Patellar position can be used as an external reference for taking a true coronal and sagittal view X-ray.

There are a few limitations in this study. The first one is that the measurements were made on 2D X-rays where the rotation of tibial component cannot be measured. Measurements on 3D CT, when compared to X-ray based measurements show better precision in such cases [13]. Measurements made in 3D CT can also be used to validate the X-ray based measurements [14]. The second limitation is that the errors which were measured may have some component involved due to inaccurate cutting by saw blade. Even if the alignment of the cutting plane is correctly determined, error can occur during the cutting process. In a few studies, cutting errors of around 1° have been reported mostly in sagittal view (compared to coronal view) because of deflection of saw blade in anterior-posterior direction [15]. Third, the study was not based on randomized control trials. However, the cutting guides were linked with the implants which were chosen on the basis of only affordability by the patient. The study was based on evaluation through postoperative X-rays, and hence the preoperative conditions of the patient were not reported. More number of cases (especially in intramedullary group) can improve the statistical power of the study for more reliable results.

## Conclusions

In conclusion, intramedullary guides used in conjunction with imported implants gave more accurate results in the coronal plane alignment of tibial component compared to extramedullary guides used with indigenous implants for megaprosthetic total knee replacement. No significant difference was found in the percentage of cases falling in the optimal range of the alignment in both coronal and saggital planes. Given the need to use tumor megaprostheses with stems in all patients, we suggest the use of intramedullary guides in all cases. Since the imported implant system is unaffordable by most of the patients, there is a need to develop an intramedullary guide for the indigenous implant. This work is currently underway.

## Abbreviations

TKR: Total knee replacement; TCA: Tibial component angle; TKA: Total knee arthroplasty; IM: Intra medullary; θ’: actual angle between anatomical axis and tibial implant axis; θ: angular measurements of tibial component angle (TCA1 or TCA2); Δ: angular error in measurements of θ; Ф: rotation of the knee.

## Competing interests

The authors declare that they have no competing interests.

## Authors’ contributions

VK participated in the design of the study, collected the study data, performed the X-ray based measurements, performed the statistical analysis and prepared the manuscript. BR contributed to the study design and edited the manuscript. MA contributed to the data collection and study design. All authors read and approved the final manuscript.
